# A Model of Alcohol Drinking under an Intermittent Access Schedule Using Group-Housed Mice

**DOI:** 10.1371/journal.pone.0096787

**Published:** 2014-05-07

**Authors:** Magdalena Smutek, Mateusz Turbasa, Magdalena Sikora, Marcin Piechota, Joanna Zajdel, Ryszard Przewlocki, Jan Rodriguez Parkitna

**Affiliations:** 1 Department of Molecular Neuropharmacology, Institute of Pharmacology of the Polish Academy of Sciences, Krakow, Poland; 2 Department of Neuroscience and Neuropsychology, Institute of Applied Psychology, Jagiellonian University, Krakow, Poland; The Scripps Research Institute, United States of America

## Abstract

Here, we describe a new model of voluntary alcohol drinking by group-housed mice. The model employs sensor-equipped cages that track the behaviors of the individual animals via implanted radio chips. After the animals were allowed intermittent access to alcohol (three 24 h intervals every week) for 4 weeks, the proportions of licks directed toward bottles containing alcohol were 50.9% and 39.6% for the male and female mice, respectively. We used three approaches (i.e., quinine adulteration, a progressive ratio schedule and a schedule involving a risk of punishment) to test for symptoms of compulsive alcohol drinking. The addition of 0.01% quinine to the alcohol solution did not significantly affect intake, but 0.03% quinine induced a greater than 5-fold reduction in the number of licks on the alcohol bottles. When the animals were required to perform increasing numbers of instrumental responses to obtain access to the bottle with alcohol (i.e., a progressive ratio schedule), they frequently reached a maximum of 21 responses irrespective of the available reward. Although the mice rarely achieved higher response criteria, the number of attempts was ∼10 times greater in case of alcohol than water. We have developed an approach for mapping social interactions among animals that is based on analysis of the sequences of entries into the cage corners. This approach allowed us to identify the mice that followed other animals in non-random fashions. Approximately half of the mice displayed at least one interaction of this type. We have not yet found a clear correlation between imitative behavior and relative alcohol preference. In conclusion, the model we describe avoids the limitations associated with testing isolated animals and reliably leads to stable alcohol drinking. Therefore, this model may be well suited to screening for the effects of genetic mutations or pharmacological treatments on alcohol-induced behaviors.

## Introduction

Animal models of alcoholism are used to investigate the mechanisms that underlie compulsive drug consumption and to test potential therapies that seek to decrease the risk of relapse. The usefulness of these models depends on their similarities to the etiologies and symptoms of addiction in humans and, more importantly, on their predictive validities. Therefore, the most widely used approaches involve alcohol self-administration, because these models appear to share the greatest similarities with the features of addiction in humans [1]–[3]. These models allow for the observation of factors that influence the acquisition of alcohol self-administration, the persistence of alcohol-associated behaviors and the mechanisms underlying relapse to drug consumption. Data obtained from preclinical models have been essential for the development of new therapies, particularly the introduction of naltrexone and nalmefene for the treatment of alcoholism [4]–[8].

A commonly used approach for modeling alcohol consumption involves the use of intermittent access schedules in which rodents with continuous access to water are also offered alcohol over three 24 h periods each week [9], [10]. The reported advantages of this approach are that it overcomes the difficulty of inducing the intake of large amounts of alcohol and that it usually produces gradual increases in alcohol intake([9]–[13]; but see [14]). Some researchers also consider the intermittent approach to be a better model of human binge-like or dependence-driven alcohol drinking [3], [15]. The effects of intermittent schedules have been observed to vary depending on mouse or rat strain, and they may be inversely correlated with initial intake levels [10], [15]. It has been proposed that the increases in alcohol consumption that are observed under intermittent schedules and after long periods of abstinence may share underlying mechanisms with alcohol-related behaviors in humans [15]. One feature of intermittent access models is that they produce results relatively quickly; these models typically produce stable alcohol intake behaviors after 2 to 5 weeks, which is of practical importance because the reduced time requirements of these models make them well suited for screening for the effects of drugs or genetic mutations on alcohol intake and alcohol-conditioned behaviors.

The existing preclinical models can reproduce the principal features of addiction, but they also have some common limitations. First, these models are designed to test isolated animals. In these models, the animal is alone in a cage during the self-administration period to simplify the measurement of drug intake and associated behaviors. Even when the self-administration sessions are conducted outside of the home cages, the animals are typically single-housed, particularly if they have been implanted with catheters or guides. Isolated mice may exhibit alterations in brain monoamine metabolism, increased food intake, higher levels of anxiety-like behaviors and aggression and altered responses to novelty [16]–[20]. Furthermore, self-administration experiments are usually performed under strictly controlled environmental conditions to reduce the number of confounding factors. Limiting the number of stimuli affects sensitivity to drug reinforcement as has been shown by the effects of enriched environments and social behaviors on alcohol drinking and drug self-administration [21], [22]. The effects of social interaction during drug access on intake and the development of addiction-like behaviors remain relatively unexplored. Notable exceptions are the recent studies on prairie voles (*Microtus ochrogaster*) that assessed alcohol drinking in isolated animals and pairs of voles that were housed in mesh-divided cages [23]–[25]. However, these studies still required that the animals be maintained in separate compartments to measure their alcohol intakes.

Here, we describe a model of alcohol drinking under an intermittent access schedule in group-housed mice that uses the IntelliCage system [26]. The main advantages of this new approach are the reproducibility of the behavioral phenotype, the ability to test the animals' motivation for obtaining alcohol in the presence of a risk of punishment and the capacity to assess the social interactions among the animals.

## Materials and Methods

### Animals

The behavioral experiments were performed on male and female mice (*Mus musculus* L.) from the C57BL/6J strain colony that is maintained at the animal facility of the Institute of Pharmacology of the Polish Academy of Sciences in Krakow, Poland. The behavioral procedures were approved by the II Local Bioethics Committee in Krakow (permit number 754/2010, issued on May 27, 2010). The mice were approximately 8–10 weeks old at the beginning of the experiments and had not been subjected to any prior testing. Before being introduced to the IntelliCages, the animals were housed 4–5 per cage in rooms with a controlled temperature of 22±2°C under a 12/12 h light-dark cycle. The animals had *ad libitum* access to standard lab chow (Labofeed H, WPiK, Kcynia, Poland) and water. The mice were killed in a CO_2_ chamber after the experiments were completed.

### Behavioral procedures

**Figure 1:**
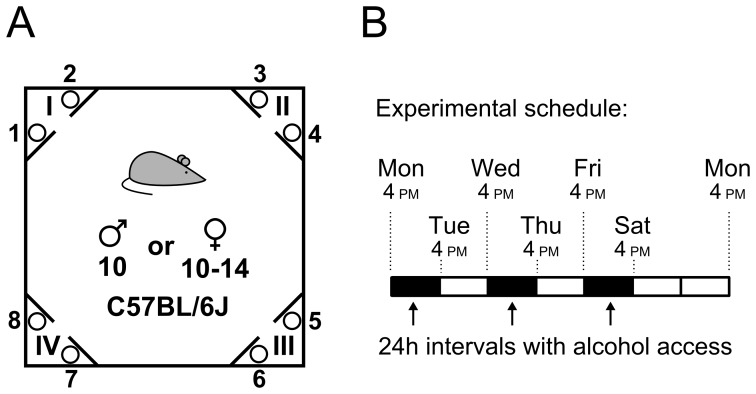
Experimental design. (**A**) The IntelliCage system. The corners are marked with Roman numerals, and the bottles are numbered clockwise from 1 to 8. (**B**) Intermittent access schedule. The bottles were exchanged six times a week at the times indicated by the dotted lines. For the last two cohorts of mice, the exchanges were performed at 3 pm. The positions of the alcohol bottles were switched after every access interval.

Behavior was analyzed using the IntelliCage system (New Behavior, Zurich, Switzerland) [26]. A diagram of the cage is shown in Figure 1A. Each corner of a cage is equipped with presence detectors, an antenna that reads RFID chip signals and a temperature sensor. Each corner also contains two photocell-equipped holes that control access to bottles containing water or another liquid. Instrumental responses toward a hole (i.e., nose pokes) opened previously closed gate and allowed the animal to drink from the bottle. The numbers of nose pokes and licks on the bottle were recorded. Additionally, the nozzles of tubes connected to tanks of compressed air located above each corner could be used to deliver air puffs to the animals' backs.

Before the experiments began, the mice were implanted with transponders (8.5 mm in length and 1.4 mm in diameter). Next, the animals were introduced to the IntelliCages in groups of 10 or 14. The mice were allowed to habituate to the cages for three days prior to the initiation of the experiments. Access to the bottles of water in the corners was either free or required an instrumental response (FR3) depending on the experimental series. All mice that lost their transponders (which prevented the behavior from being recorded) received replacement transponder chips.

#### Intermittent alcohol access procedure

The intermittent alcohol access schedule was adapted from the literature [9], [10] and is outlined in Figure 1B. The mice were provided access to 4 bottles (in 2 corners) that contained alcohol during the following three 24 h periods every week: 4 pm Monday to 4 pm Tuesday, 4 pm Wednesday to 4 pm Thursday and 4 pm Friday to 4 pm Saturday. During the last series of experiments (i.e., the last two cohorts), the bottles were exchanged at 3 pm. The corners that contained the alcohol were switched after each session; corners I and III contained alcohol during the odd-numbered sessions, and II and IV contained alcohol during the even-numbered sessions. The concentrations of alcohol were 4% (v/v) during the first session, 8% during the second session and 12% in all subsequent drinking sessions.

The first phase of experiments consisted of 12 or 15 alcohol access sessions, which corresponded to 4 or 5 weeks in the cage. Access to the bottles was either free or required an instrumental response to raise a gate that barred access to the bottle. The instrumental task required three nose pokes (FR3). There was no set maximum interval between the nose pokes, and there was no time limit to complete the task, but the criterion had to be met within one continuous corner visit. The gate opened for 5 seconds after the third nose poke. Additional nose pokes had no consequence. When the gate closed, the animal was required to leave and re-enter the corner before a new FR3 task could be completed. The numbers of individual visits, drinking events and bottle licks were calculated as sums for each bottle and corner that contained either alcohol, saccharine or water.

#### Quinine adulteration

A schematic outline of the adulteration procedure is shown in Table S1. The quinine adulteration procedure was performed on a cohort of 10 male mice after 5 weeks (15 sessions) of free alcohol access. During the subsequent 24 h sessions, the animals had access to water in two of the corners, and the remaining two corners contained bottles that contained the following: (i) 12% alcohol (v/v) and 0.02% (w/v) saccharin in opposite corners, (ii) 0.02% saccharin + 0.01% quinine in both corners, (iii) 12% alcohol adulterated with 0.01% quinine in both corners, (iv) 0.02% saccharin with 0.03% quinine in both corners, (v) 12% alcohol with 0.03% quinine in both corners, (vi) 0.02% saccharin with 0.03% quinine in both corners, or (vii) 12% alcohol with 0.03% quinine in both corners. The positions of the water and alcohol/saccharine bottles were switched after each 24 h session (see Table S1).

#### Progressive ratio instrumental schedule

The progressive ratio (PR) schedules required the animals to perform increasing numbers of operant responses to obtain access to bottles containing 12% alcohol, 0.02% saccharin or water. The number of required responses was increased by 1 (PR1) or 3 (PR3) each time the animal gained access to the bottles. Testing was performed in two series of experiments, which are outlined in Figure S1. The first series of experiments was performed on a cohort of 10 male mice after 5 weeks (15 sessions) of intermittent alcohol access. During the subsequent 24 h sessions, the animals had access to water in two of the corners, and the remaining two corners had bottles that contained the following: (i) 12% alcohol (v/v) and 0.02% (w/v) saccharin in opposite corners, (ii) 0.02% saccharin under a PR1 schedule (both corners), (iii) 12% alcohol under a PR1 schedule (both corners), (iv) 0.02% saccharin under a PR3 schedule (both corners), or (v) 12% alcohol under a PR3 schedule (both corners). The positions of the water and alcohol/saccharine bottles were switched after each 24 h session (see Figure S1).

The second series of experiments was performed on 3 cohorts of 10 female mice. The first cohort was provided with intermittent operant access to alcohol during 12 sessions over 4 weeks. The second cohort was provided intermittent access to 0.02% saccharin under a schedule that was identical to that of the first cohort. The third cohort was provided with access to water bottles only. After the intermittent access period, the mice were tested in three additional 24 h sessions with the following reinforcement contingencies: (i) FR3 access to water in two corners and alcohol/saccharin/water under a PR1 schedule in the remaining two corners (i.e., the contingencies were identical to those of the intermittent access period); (ii) FR3 access to water in all corners, and (iii) FR3 access to water in two corners and alcohol/saccharin/water under a PR3 schedule in the other two corners. There was no time limit to perform the PR task, but the task had to be completed within one visit to a corner.

#### Drinking despite the risk of punishment

Experiments modeling the drinking of alcohol or saccharin under the risk of punishment were conducted on cohorts of male mice that had completed the PR testing. The mice underwent three sessions that included the risk of punishment. The first session involved FR3 access with 100% risks of punishment for 12% (v/v) alcohol in one corner and for 0.02% (w/v) saccharin in the opposite corner along with FR3 access without punishment to water bottles in the remaining two corners. The second and third sessions involved FR3 access with 25% risks of punishment for 12% (v/v) alcohol in one corner and for 0.02% saccharin in the opposite corner along with FR3 access to water without a risk of punishment in the remaining corners. The punishment consisted of a 0.5 bar air puff that was delivered to the animals' backs 2 s after the FR3 was completed.

In a second series of experiments, two cohorts of female mice that had completed PR responding for alcohol or saccharin were provided with intermittent access to a reward for 1 week. A third cohort of female mice received continuous access to water only. Next, a single testing session was performed during which the animals were provided with FR3 access to alcohol, saccharin or water associated with a 25% risk of punishment in two corners and FR3 access to water without punishment in the remaining two corners. The punishment consisted of a 0.5 bar air puff that was delivered to the animal's back 2 s after the FR3 was completed. Figure S1 shows schematic outlines of the punishment risk procedures.

### Data analysis

The raw results from the cages' sensors were stored as plain text tables containing all recorded events (e.g., corner visits). The tables were parsed using internally developed tools for the JAVA and R platforms [27]. The extracted data were analyzed using R and Graphpad Prism. Statistical analyses of the differences in mean values were performed with analyses of variance (ANOVAs) followed by *post hoc* tests or Student's t-tests where appropriate.

Analyses of social interactions were performed on data extracted from the last 4 intervals of alcohol access. Each mouse was assigned a letter of the alphabet, and his/her sequence of entries into the corners (each corner separately) was converted into a string of characters. The occurrence of letter pairs in each string was then counted, and the cases in the time elapsed between visits was shorter than 1 s or longer than 1 minute were excluded. To assess whether the frequency of pair occurrence diverged from a random distribution, the initial strings were randomly permuted 100 times each, and the mean frequencies and standard deviations were calculated.

## Results and Discussion

### Behavioral activity in the IntelliCages

**Figure 2:**
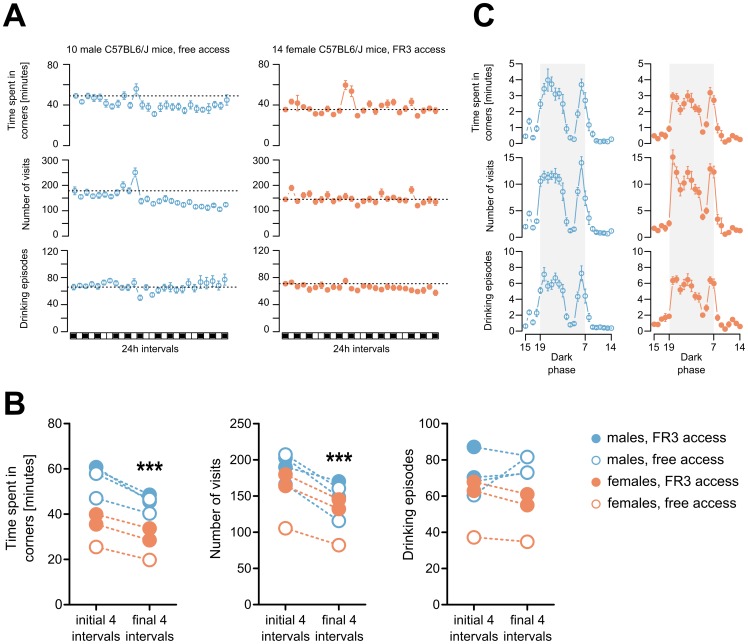
Corner activity during the experiment. (**A**) Activity in the cage corners during the experiment. The graphs illustrate the mean daily time spent in the corners and the numbers of visits and drinking episodes. Two series of data are shown; the first is for a cohort of 10 male mice with free access to bottles, and the second is for a cohort of 14 females with FR3 instrumental access. Each of the boxes on the bar below corresponds to a 24-h period, and the black boxes correspond to periods of access to alcohol. The dashed horizontal lines correspond to the value on the first day. (**B**) Circadian activities of the animal groups shown in A. The graphs illustrate the mean patterns of daily activity of the mice averaged across four intervals (7th, 14th, 21st and 28th). Each point represents the sum from the preceding hour. The dark period is shaded grey. (**C**) Summary of the corner activity data. The graphs illustrate the mean daily activities averaged across the first and last 4 days of the experiment. The dashed lines connect the points that correspond to the same cohort. The error bars in panels A and B represent the SEM. The significance of the differences in behavior during the final 4 days compared to the initial 4 days in panel C was calculated using a paired t-test (P<0.001 ***). The data are summarized from all experiments, which included 4 cohorts of 10 male mice, 2 cohorts of 14 female mice and 1 cohort of 10 female mice.

The intermittent alcohol drinking procedure in the IntelliCages was tested on seven cohorts of C57BL/6J mice composed of four groups of 10 males, two groups of 14 females and one group of 10 females. Larger numbers of females (14) were tested to observe their behavior in groups of sufficient size to include two subgroups that could differ in genotype, rearing conditions or pharmacological treatment. Additionally, two control experiments were performed. The first control consisted of 10 females with intermittent access to a 0.02% w/v saccharin solution under an FR3 schedule. The second control experiment was performed on a group of 10 female mice that had FR3 access to water only. These controls were intended to compare the effects of a “natural” reward or no additional reward with those of alcohol. The general behavior of the animals during the first phase of the procedure is summarized in Figure 2. Whether the access to the bottles was operant-dependent or free did not affect the mean times the mice spent in all corners (Figure 2A). The mean daily times spent in the corners and the numbers of corner visits decreased over the course of the experiment in each experimental cohort (Figure 2B). There was a trend toward shorter mean times spent in the corners by the female cohorts compared to the male cohorts, which may reflect a difference in territorial behaviors. Conversely, the mean number of drinking events was similar across all groups throughout the experiment. The circadian patterns of activity (as observed from the corner visits) peaked in the first part of the dark phase and peaked again before the light phase (Figure 2C), which is consistent with a previous report where single-housed C57BL/6J mice were studied [28]. Activity during the light phase was minimal. There were no significant effects of sex, group size or instrumental access on circadian activity. These data show that the general behaviors of all of the mouse cohorts were consistent with the exception of apparently minor differences between the males and females.

We also examined whether the mice tried to nest in the corners and thus prevent the other animals from accessing them. The majority of the corner visits were less than 1 minute in duration and the durations of relatively few visits exceeded 10 minutes. Examples of the distributions of corner visit durations over a 4-week period are shown in Figure S2. These data also show that the intervals between visits by the same mouse to the same corner were generally >10 s and were widely distributed. These findings suggest that the technical artifacts that may have resulted from loss of contact between the implanted chip and the detector in the cage corner were most likely uncommon.

**Figure 3:**
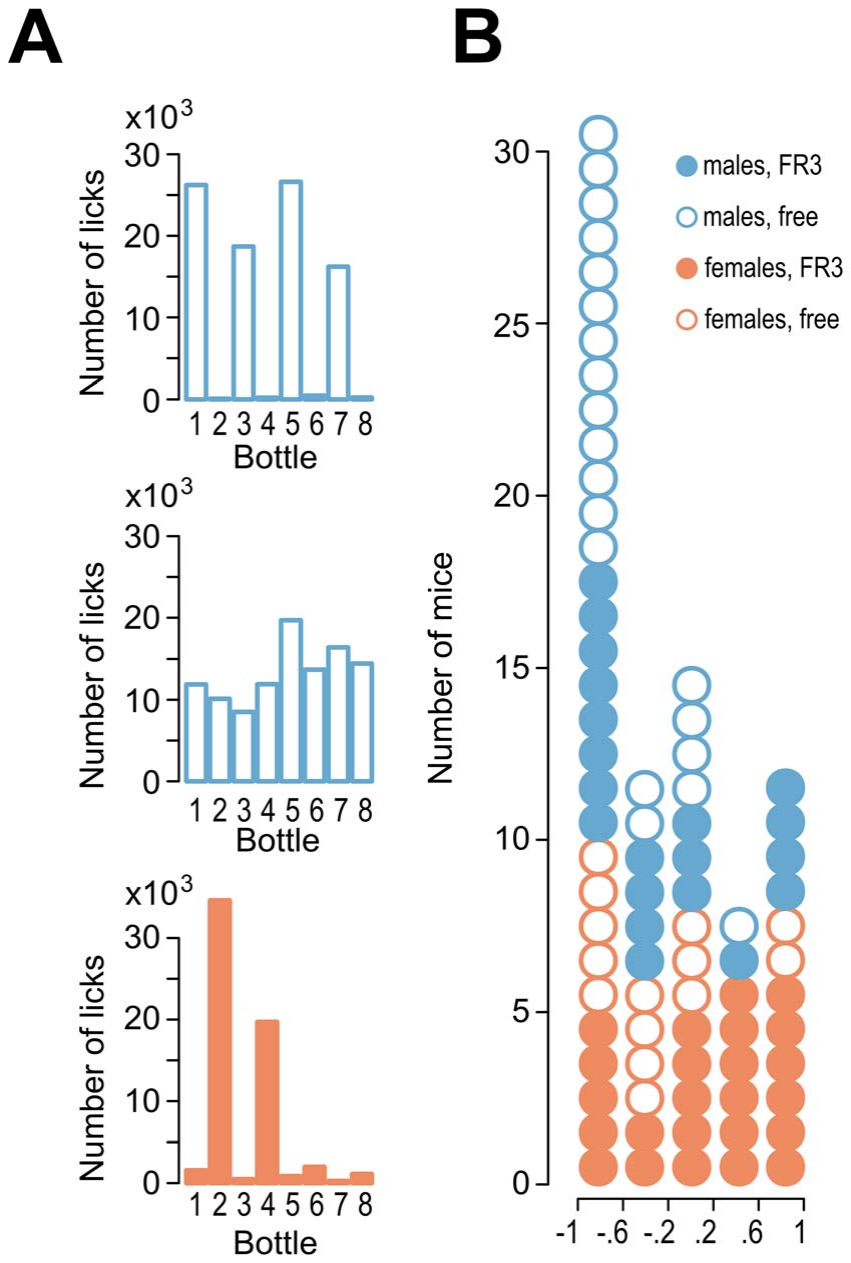
Side-preference bias. (**A**) Examples of individual mice that exhibited biased or unbiased side choice. The graphs show the sums of the licks on individual bottles during the intermittent access experiment. The top graph is an example of a mouse with a strong preference for the right side, the middle graph corresponds to an animal without a choice bias and the bottom graph shows an animal with a left side bias and a corner bias. (**B**) The frequencies of side biases. Each circle on the histogram represents a single animal. Preferences values were calculated as follows: ([right side licks – left side licks]/[right side licks + left side licks]). The summary in panel B includes all mice that were tested for alcohol drinking (40 males and 38 females).

We frequently found biases in the choices between two equivalent bottles in the same corner. The majority of mice primarily drank from one side of the corner. Examples of animals with strong left biases, no biases and right side biases in corner preference are shown in Figure 3A. Among the 78 mice tested, 31 showed strong (<−0.6) preferences for the left bottle, and 12 showed strong (>0.6) preferences for the right bottle (Figure 3B). This effect was not dependent on the type of access (FR3 compared to free) and was observed in both male and female mice. It is unlikely that this effect was caused by environmental cues that were present in the room (e.g., lights, computer noise) because it was observed in all experimental cages, and even in a different room. We conclude that the biases in bottle choice may reflect inherent lateralization of this behavior.

### Intermittent alcohol drinking

**Figure 4:**
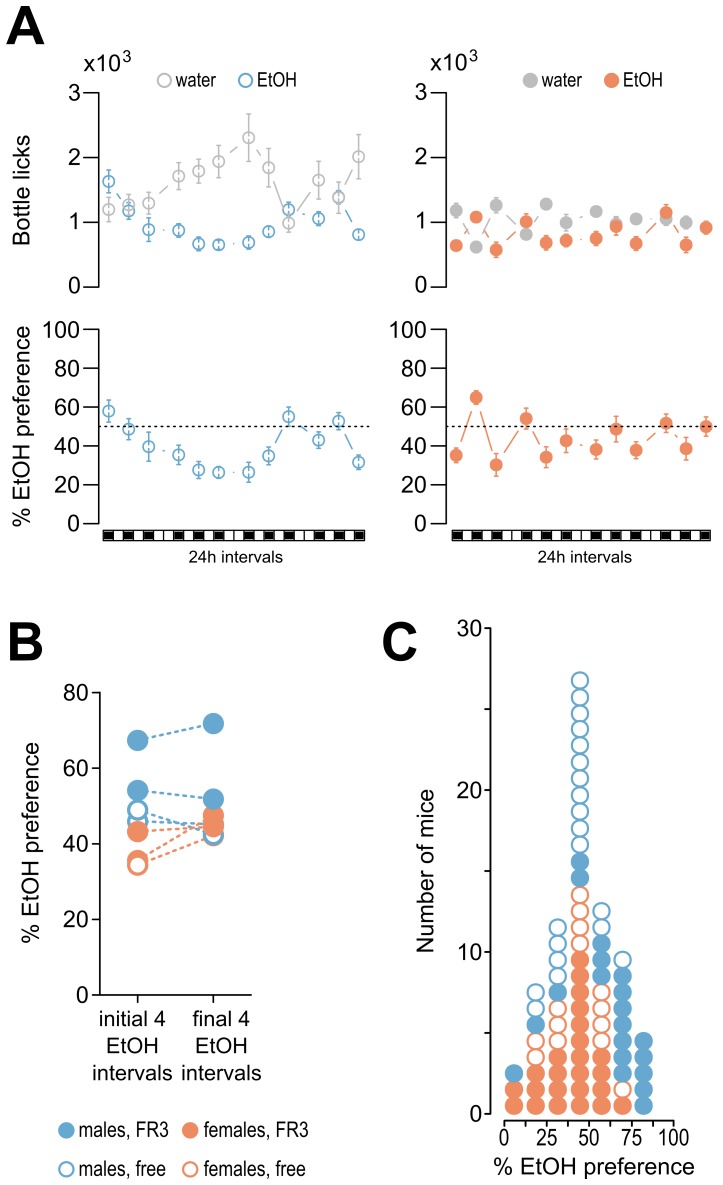
Alcohol preference. (**A**) The choice between bottles containing alcohol or water. The top two graphs show the mean numbers of licks on the bottles with 12% alcohol or tap water. The left graph is from a cohort of 10 males with free access to the bottles and the right graph is from a cohort of 14 females with FR3 instrumental access. The two lower graphs show the corresponding ratios of alcohol preference, which were calculated as follows: ([alcohol licks]/[alcohol licks + water licks]). (**B**) Summary of the alcohol preference results. The graph shows the averaged preferences over the first 4 intervals with alcohol access compared to those of the last 4 intervals. The data points corresponding to the same cohort are connected with dashed lines. (**C**) Variation in alcohol preference. The histogram shows the individual alcohol preferences of all mice tested averaged over the final 4 access intervals. Each circle corresponds to a single mouse. The distribution of values did not diverge from normal (Shapiro-Wilk's test W = 0.9826, P = 0.3653). Nevertheless, the mean preferences differed between the males and females (50.9±2.8% and 39.6±2.5%, respectively, t-test P = 0.0041).

The mice were given intermittent alcohol access over four weeks or, in one experiment, over five weeks. The mean alcohol preference, which was defined as the ratio of licks on the ethanol bottles divided by the total number of licks on all bottles, during the first and last four intervals of alcohol access was similar across the 7 cohorts tested (Figures 4 A&B). We estimated that a single lick on a bottle corresponded to the consumption of 3 µl of liquid. For the cohort of male mice shown in Figure 4A, the mean number licks on the 12% v/v alcohol bottle per day during the last two intervals of access was 1257±148, which corresponds to ∼0.436 g of EtOH per interval. Assuming a mean animal weight of 26 g (based on measurements performed after the experiment was finished), the alcohol dose 16.8 g/kg/24 h over the last two intervals for the male cohort shown in the example. This value is in the lower range of those that have been reported (15–30 g/kg/24 h) in studies that have been performed on single-housed mice with intermittent alcohol access (e.g., [11], [15]). We have previously determined that ∼300 licks over 1 h corresponds to a blood ethanol concentration of 0.1‰ w/v, which is equivalent to the levels observed 20 minutes after 1 g/kg i.p. injections of ethanol in mice from the same colony [29].

No increase in alcohol intake over subsequent sessions was observed, and this finding differs from those of some previous reports [9]–[11] but is consistent with another report [14]. The mean level of alcohol preference observed in all male mice (50.9±2.8%) was lower than that previously reported (approximately 65% in C57BL/6J mice with 4–6 weeks of intermittent access to alcohol [11], [15], 68–75% under continuous access to alcohol [30]–[32] and over 80% after 3 months of continuous access in IntelliCages [29]). The relative alcohol preference among the group-housed male mice was higher than that of the group-housed females in the present experiment (50.9±2.8% compared to 39.6±2.5%, respectively, t-test p = 0.0041). Moreover, a two-way ANOVA indicated that, in addition to an effect of sex (F_1,74_ = 9.946, P<0.01), there was also a significant interaction between sex and access type (F_1,74_ = 8.489, P<0.01) and a trend toward a higher relative alcohol preference during instrumental access (F_1,74_ = 3.943, P = 0.05076). The latter effect was somewhat expected because the rewarding of operant responses was likely followed by conditioned reinforcement, which could have increased alcohol drinking. However, the effect was apparently present only in the male mice. When the factor of sex was excluded, the effect of the type of access was relatively small and did not reach significance (47.9±3.1 vs. 42.37±2.2). This potential interaction between sex and type of alcohol access was surprising, and we were unable to find an existing report containing a similar observation. Furthermore, we found that the male mice drink higher proportions of alcohol compared to water than did the females; this finding differs from those of some previous reports (e.g., [32]). We speculate that this finding could be related to sex differences in the sensitivity to isolation because isolation-induced increases in anxiety or stress may alter relative alcohol preferences [33]; although it should be noted that the C57BL/6J strain is often regarded as stress-resistant (e.g., [19]).

We did not find evidence that the mice worked harder to obtain alcohol. There was no increase in the time spent in the cage corners or in the numbers of corner visits when alcohol was available (Figure 2A). We also found that, in general, greater reward preferences were not necessarily correlated with increased effort. The mice given intermittent access to saccharin consistently exhibited preferences greater than 80%, but the total volumes of liquid consumed (i.e., bottle licks) and corner activities were not affected (Figure S3). Individual alcohol preferences ranged from 0% to 80% (Figure 4C). The distribution of individual preferences did not diverge from normal. There was no indication of a multinomial distribution (i.e., there was no defined subpopulation of mice with a high relative alcohol preference). We did not find significant correlations between alcohol preference and animal weight, activity or bottle-side bias (all Pearson's r^2^<0.2, n.s.).

### Effects of adulteration, progressive schedules and punishment risk on alcohol drinking

Animal models of addiction emphasize behaviors that may reflect compulsive drug consumption. Three approaches are commonly used. The first involves the adulteration of alcohol with a bitter taste (quinine) and models the persistence of alcohol intake despite degradation of the value of the outcome. The second approach employs progressive ratio (PR) schedules of instrumental responding that require increased effort to obtain alcohol. The third approach pairs alcohol intake with punishment to model the consumption of alcohol despite negative consequences.

**Figure 5:**
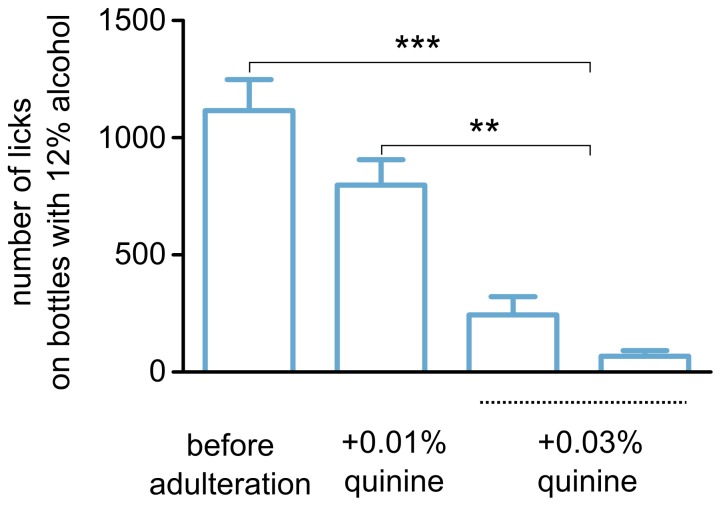
Effects of adulteration with quinine. The bar graphs show the mean numbers of licks on the bottles with 12% alcohol adulterated with increasing concentrations of quinine of a cohort of 10 male mice (see Table S1 for a summary of the experiment). Each bar corresponds to a single 24-h interval, and only intervals with access to alcohol are shown. Analysis of variance indicated that the means were significantly different (F_3,56_ = 26.54, p<0.001; Tukey's HSD *post hoc* P<0.01 ** and P<0.001 ***). There was no significant difference between mean numbers of licks on the alcohol bottles before adulteration and after adulteration with 0.01% quinine.

The effects of the adulteration of alcohol were tested on mice that previously had free intermittent access to alcohol bottles. Adulteration via the addition of 0.01% (w/v) quinine decreased the mean number of licks on the alcohol bottles, but this effect was not significant (Figure 5). A further increase in the concentration of quinine to 0.03% significantly reduced alcohol intake compared to both the initial levels and the levels observed after 0.01% quinine adulteration. These results indicate that concentrations of quinine between 0.01 and 0.03% may be adequate to measure the propensity to consume alcohol despite reduced value. A comparison of effects of quinine adulteration on saccharine vs. alcohol drinking is shown in Figure S4.

**Figure 6:**
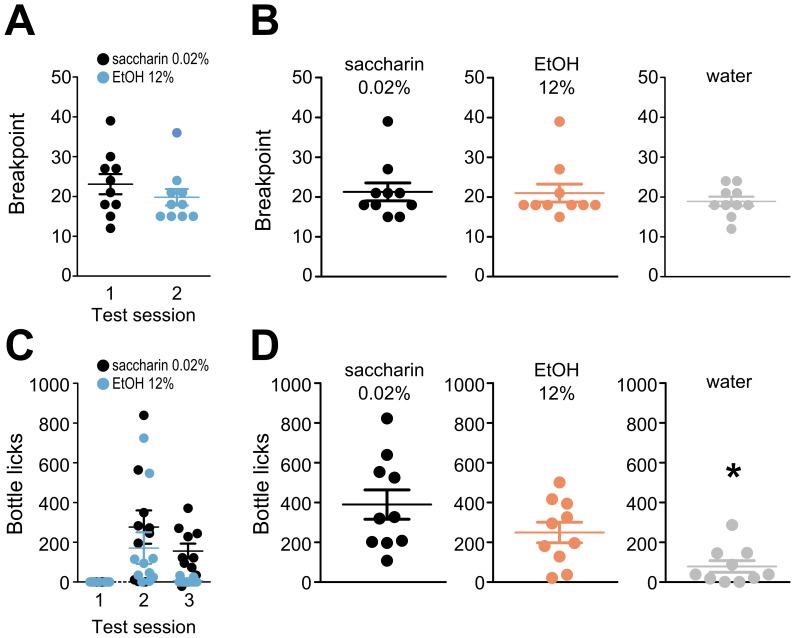
Instrumental responding under a progressive schedule or a risk of punishment risk. (**A, B**) Instrumental responding under a progressive ratio (PR3) schedule was elicited as described in the Methods. The graphs show the breakpoints reached by each individual animal. The horizontal bars indicate the group mean and SEM. Panel A shows the results for a group of 10 male mice that was sequentially tested under PR3 schedules of access to saccharin and ethanol. Panel B represents 3 separate groups of 10 females each that were tested under PR3 schedules of access to saccharin, alcohol or water. (**C, D**) Instrumental responding with the risk of punishment. The mice were provided with FR3 instrumental access to alcohol, saccharin or water. Panel C shows the numbers of bottle licks performed by individual male mice (the same cohort shown in A) that were tested in separate intervals of access to saccharin or alcohol. During the first session, there was a 100% risk of punishment immediately after the FR3 was completed. During the second and third sessions, the punishment risk was 25%, and punishments were delivered 2 seconds after completing the FR3. Panel D shows the numbers of bottle licks performed by the individual mice of three cohorts that were separately tested with a 25% risk of punishment delivered 2 seconds after the FR3 was completed; these are the same cohorts of mice shown in panel B. The horizontal bars indicate the group means and SEMs. The statistical analysis of the group means in Panel D was performed via one-way ANOVA (F_2,29_ = 8.188 P<0.01, Tukey's HSD *post hoc* P<0.05 * saccharin vs. water).

PR schedules are often used to measure the motivation to obtain reward. The classical procedure involves testing a single animal in a Skinner box in which the animal has access to two operants [2], [34]. The animal has previously been trained to recognize that an instrumental response on one operant has no consequence, while a response on the other has been associated with a specific outcome. The number of instrumental responses required is progressively increased during the PR testing until the animal no longer completes the task, i.e., until the animal reaches its “breakpoint”. We employed a similar approach to test the behaviors of mice that had previously been trained under an FR3 schedule (see Figure S1 for the full experimental schedule). These mice usually reached their breakpoints at approximately 21 instrumental responses (Figures 6A&B) when tested with alcohol alternating with saccharin (Figure 6A) or when the mice were exposed to only one type of reward (Figure 6B). Surprisingly, the same breakpoint levels were reached by the mice that only had access to water and were able to choose between PR and FR3 water-access schedules during a session. However, closer examination of the data revealed differences in performance on the PR3 schedule (Figure S5). For example, the numbers of visits to the corners containing saccharin or alcohol after the breakpoints were reached were higher (mean ± SEM: 89.5±13.56 for saccharin and 61.7±13.33 for alcohol) than those in cages in which only water was available (6.3±3.06). We interpret this difference to be the result of continued failed attempts to obtain saccharin or alcohol and a lack of continued effort to obtain water. Thus, under our experimental conditions, the breakpoint was not a measure of motivation. However, analyses of the numbers of corner visits during PR-schedule access indicated possible differences in the motivations to obtain saccharine or alcohol compared to water.

The propensity to drink despite the risk of punishment was measured in the mice that were given access to alcohol under an FR3 schedule. The punishment consisted of a 0.5 bar air puff that lasted 0.2 s and was delivered via a tube located above the corner. The air puff was not harmful but was highly aversive. The mice did not drink from the available bottles when the air puffs were delivered immediately after the animals had reached the FR3 criteria (Figure 6C), and similar results were obtained for the 12% alcohol and 0.02% saccharin solution reinforcers. We delayed the air puff delivery until 2 s after the moment the bottle access door opened to associate the punishment with the reward rather than with the instrumental response. Additionally, the probability of the punishment was decreased from 100% to 25%. Under these conditions, the mice drank from the bottles despite the risk of punishment; however, this behavior exhibited considerable inter-individual variability (Figure 6C). There were significantly fewer licks for water than for saccharin among the animal cohorts that were allowed access to only one type of reward, and a similar difference was observed when licks for water vs. alcohol were compared (Figure 6D). These results indicate that the mice were willing to risk a punishment to obtain a reward. Whether this willingness represents compulsivity is a matter of interpretation. The ability to evaluate the trade-off between reward and punishment is essential for survival. The behavior observed during this experiment may reflect normal behavioral adaptability rather than an inability to refrain from seeking the reward. Nevertheless, this test appears to be well-suited to assess the effects of genetically engineered mutations or pharmacological treatments on the ability to evaluate the trade-off between reward and punishment, which is relevant for the treatment of addiction and impulsive disorders.

### Social behavior

**Figure 7:**
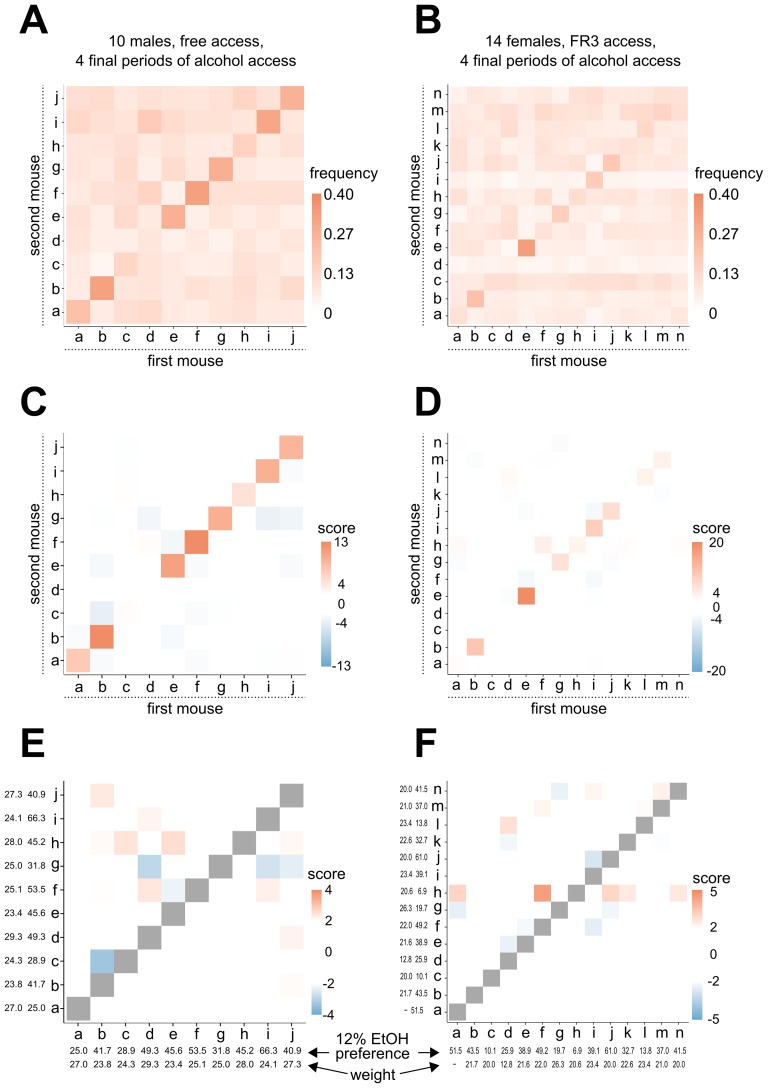
Social interactions of the mice. (**A, B**) Frequencies of sequences of entries into the corners. The heat maps show the relative frequencies of combinations of visits into the same corner by a pair of mice that occurred within the interval of 1 second to 1 minute. Each mouse was assigned an alphabet letter. On the maps, the first mouse in the sequence is indicated on the x-axis, and the second mouse is indicated on the y-axis; e.g., the bottom leftmost square corresponds to the frequencies of the ‘aa’ pairs. The intensity of the color is proportional to fraction of a specific pair among all pairs with the same first mouse (thus each column has a sum of 1). The intensity scales are indicated to the right of each heat maps. The data shown correspond to the last 4 intervals with alcohol access. Panel A shows data derived from 2972 pairs (filtered from 6548) and panel B represents 4643 pair sequences (filtered from 8573). (**C, D**) Interactions diverging from a random pattern. The heat maps show the relative statistical significances of pairs of entries (see Methods for the score calculation method). Only pairs with |scores| >2 are shaded. (**E, F**) Interactions diverging from a random pattern with repeats removed. The scores were calculated as in C and D, but they exclude all instances of the same mouse performing repeated entries (e.g., ‘bb’). The numbers shown near the axes are the alcohol preferences and animal weights. Only pairs with |scores| >2 are shaded.

We analyzed the animals' sequences of entries into each of the corners during the last 4 alcohol access intervals to determine whether social interactions influenced alcohol-drinking patterns (Figure 7). Our reasoning was that, if the mice observed each other's behavior, then they would enter or avoid entering a corner after another animal had visited that corner. The first step of the analysis was to establish the frequencies of the sequences of mouse entries (Figure 7 A&B). However, the frequencies alone are not sufficient to identify pairs that diverge from the random distribution. Therefore, we calculated the differences between observed frequencies and theoretical frequencies that were generated via random permutation of the data set (Figure 7 C&D). This analysis revealed that the most common pattern involved individual mice visiting the same corner multiple times in sequence. This pattern was likely due to natural exploratory behavior, but we also considered the possibility that it resulted from technical artifacts in which the detectors lost and reestablished contact with the implanted radio chip. Although we considered this possibility unlikely (Figure S2), we performed a parallel analysis in which all strings of visits by the same mouse were removed (Figures 7E&F). Assuming that the frequencies were normally distributed, scores with values greater than 2 or smaller than -2 would indicate unadjusted P values of 0.0455. Values greater than 3.5 would indicate P values below 0.0005. However, if the observed frequencies diverged from the normal distribution and were not independent, the scores would only approximate estimates of statistical significance.

Keeping the limitations of this analysis in mind, several conclusions may be drawn. First, some of the animals tended to enter the same corner repeatedly. This effect had the highest observed significance. Second, the mice rarely followed the behavior of other mice when visiting corners (there were few results with |scores| >3.5). The frequencies of the cases of following or avoiding another animal were approximately equal across the male and female mice. The relationships were rarely reciprocal; e.g., mouse “*h”* followed *f*, but “*f”* did not follow (or avoid) “*h”* (Figure 7F). Furthermore, no correlation was found between the relative preferences for alcohol within the associated mouse pairs. Mice with very low relative alcohol preferences often followed animals with high preferences and vice versa; e.g., animals “*h”* and “*f”* in Figure 7F and “*h”* and “*c”* in Figure 7E, respectively. Finally, there was no discernible pattern in the body weights (a factor influencing social dominance) among the correlated mouse pairs as measured at the end of the experiment. This result partially agrees with those of studies of alcohol intake in socially housed prairie voles in which the influence of one animal's alcohol preference on the preference of another animal was dependent on sex [24], [25].

It is not possible to conclude whether these results were to be expected because there is no published account of a similar analysis of the social networks of group-housed mice. We should note that several mice exhibited no apparent tendency to follow any other animal (e.g., mice “*a”* and “*b”* in Figures 7E&F). In some cases, a single mouse followed several others (mouse “*h”*, Figure 7F), and we speculate that this behavior reflected dominance. Following or avoiding other mice in corner visits is certainly not representative of all of the social behaviors that occurred the group, but following and avoiding behaviors have two particularly interesting features. First, these behaviors are not ambiguous and can be counted automatically. The number of interactions that can be analyzed is limited only by the size of the group in the cage. These interactions are natural and not in any way forced on the animals by the design of the experiment. Nearly all previous models have relied on an observer who scores the types of social interactions. These types of models typically involve animals that are selected arbitrarily and are tested outside their home cages ([35]–[37], but see [23], [38] for notable exceptions). A second important aspect of our model is that it can be interpreted in terms of the influence of social interaction on behavioral choice. Our model can easily be adapted to include outcomes of corner visits that are specific to each mouse and thus could be used to examine how differences in outcomes affect the relationships between mice. To our knowledge, this is the first method that permits the mapping of the social networks of mice and the effects of those social networks on reward-induced behaviors.

## Conclusion

Our model of intermittent alcohol drinking in group-housed mice shows that it is possible to assess reward-driven behaviors in a group of animals without isolating them or simplifying their environment with minimal interference from the experimenter. We found that, after 4 weeks of intermittent access, alcohol was still consumed despite adulteration (0.01% quinine) or the risk of punishment, but the control experiments suggested that the observed behaviors were not necessarily forms of compulsive alcohol drinking. Longer periods of drug access are likely required before addiction-like phenotypes develop. This supposition is supported by the results of two other recently published studies using IntelliCages in which the phenotypes were tested after extended periods (i.e., several months) of alcohol drinking [29], [39]. In general, the relatively short time required to assess behavior and the ability to test a cohort of mice together in the same cage make this new model well-suited to screening the effects of genetic mutations on alcohol drinking.

Compared to previously reported data, we found that group-housed mice readily drank alcohol, although the mice exhibited lower intakes than those previously reported for isolated animals. We speculate that this difference may have been caused, to some extent, by the effects of isolation on the activity of the serotonin system. Analyses of rodents that have been selectively bred for high alcohol preference have revealed altered serotonin signaling and lower brain serotonin content in these alcohol-preferring rodents [40]–[42]. Conversely, it has been observed that individual housing of mice is associated with altered serotonin metabolism and decreased availability of the 5-HT_1A_ serotonin receptor [43], [44]. Thus, social insolation may affect serotonin metabolism and signaling and influence alcohol intake. The circadian patterns of activity were also notable. The mice in the IntelliCages were active almost exclusively during the dark phase during which they exhibited two peaks of activity that were separated by a two-hour period of very low activity. This pattern is similar to the previously reported in single-housed C57BL/6J mice [28] but differs from those expressed by mice that have been selectively bred for high or low alcohol intake in the “drinking in the dark” (DID) procedure and animals that have been selected for sensitivity to withdrawal-induced seizures [45]; the wheel-running activity patterns of these animals do not exhibit the two-peak pattern. Finally, we found that male mice exhibited greater relative alcohol preferences than did the female animals, and the male mice also increased their intake when they were required to perform an operant response to receive access to the reinforcer. The greater relative alcohol preference of the males contrasts with some observations of individually tested mice (e.g., [31]). Moreover, we did not find evidence of a split between “non-alcohol-preferring” and “alcohol-preferring” mice. Although the individual relative alcohol preferences were highly variable (0% to 80%), the total distribution of individual preferences did not diverge from normal. However, because an effect of sex and a possible effect of schedule were found, subtle divergences from normality may have been obscured.

Here, we developed a simple methodology for the analysis and visualization of social interactions and the identification of imitative behavior. However, we found that instances of mice visiting or avoiding corners that other mice had already explored were relatively infrequent and that only a few mouse pairs per cohort exhibited strongly correlated behaviors. Further tests are required to draw conclusions about the potential effects of social interactions on alcohol-driven behaviors.

## Supporting Information

Figure S1 **Schedules of the progressive ratio and punishment risk experiments**. The diagrams show the schedules of access to saccharine or alcohol. Each box represents a 24-h session. The instrumental schedules (FR3, PR1, PR3) or added risks of punishment (air puff) are indicated inside the boxes. The arrows illustrate the availability of 12% v/v alcohol or 0.02% w/v saccharine in the corners indicated by the labels. (**A**) The sequence of the tests that were performed on a cohort of 10 male mice that were allowed intermittent access to alcohol for 4 weeks. The experimental schedule corresponds to data shown in panels A&C of Figure 6. (**B**) The sequence of tests that were performed on 2 separate cohorts of 10 female mice that received 4 weeks of intermittent access to alcohol or saccharine (0.02% w/v) and were subsequently tested under a progressive ratio schedule followed by drinking despite the risk of punishment (25% probability of an air puff delivered 2 s after the FR3 was completed). (**C**) The sequence of tests that were performed on a cohort of 10 female mice that had only continuous access to water. The experimental schedule shown in B&C corresponds to panels B&D of Figure 6.
(TIF)

Figure S2 **Durations of and intervals between corner visits.** The upper two histograms show the distributions of the durations of all corner visits during the 4-week procedure in two representative cohorts of mice (i.e., a group of 10 males and another groups of 14 females). The scales of the axes are logarithmic. The lower two histograms show the distributions of the time intervals between two consecutive visits of an animal to the same corner. The histograms show that most of the visits were > 3 s in duration and that the intervals between consecutive visits were typically > 3 s. While a fraction of the very short times may have resulted from occasional, temporary losses of contact between the cage on the RFID chip in the mouse, such events are unlikely to have significantly contributed to the data.
(TIF)

Figure S3 **Corner activity and drinking preference of a cohort of 10 male mice with intermittent access to 0.02% saccharin.** (**A**) Mean daily time spent in the corners, numbers of visits and drinking episodes. (**B**) The numbers of licks on the saccharin or water bottles and saccharin preferences.
(TIF)

Figure S4 **Effects of adulteration with quinine.** The bar graphs show mean numbers of licks on bottles with 0.02% saccharin or 12% alcohol adulterated with increasing concentrations of quinine during a single interval of free access. The procedure was carried out over 6 subsequent intervals. Alcohol and saccharin testing was performed during separate intervals, as described in the Methods (see Table S1 for a summary of the experiment). Repeated measures ANOVA F_5,59_ = 19.35, p<0.001; Tukey's HSD *post hoc* comparisons of 1 vs. 2, 3 vs. 4 and 5 vs. 6 were not significant.
(TIF)

Figure S5 **Individual breakpoints of the mice that received PR3 instrumental access to saccharin, alcohol or water.** The data shown on the graph represent the same cohorts of mice as shown in Figure 6B. (**A**) The heights of the bars correspond to the breakpoints of the individual mice. Each box corresponds to a completed PR stage. The filled boxes indicate that the animal licked the bottle after reaching the criterion. The open boxes indicate that no licks were detected (and presumably, the reward was not consumed). The number inside the box indicates the number of visits the mouse performed before reaching the next criterion. The top boxes represent PR criteria that were not reached and are therefore always empty. (**B**) The graphs show the mean number of visits performed in the “rewarded” corners after the breakpoint was reached (left) and the mean number of visits performed between completion of the PR criteria (right). In both cases, analysis of variance indicated the presence of a significant difference across groups (F_2,27_ = 14.52, P<0.001 and F_2,27_ = 6.043 P<0.01, respectively). Significant differences between the mean values of the visits were calculated using Tukey's HSD; “*” corresponds to P<0.05 vs. “water”, “**” P<0.01 and “***” P<0.001. There were no significant differences between the mean numbers of visits by the “alcohol” and “saccharin” groups.
(TIF)

Table S1 **Quinine adulteration schedule.**
(DOCX)
