# Supplementary figures and images for: A Model of Alcohol Drinking under an Intermittent Access Schedule Using Group-Housed Mice
Source: PLoS One. 2014 May 7;9(5):e96787. doi: 10.1371/journal.pone.0096787 (PMC4013044; doi:10.1371/journal.pone.0096787)

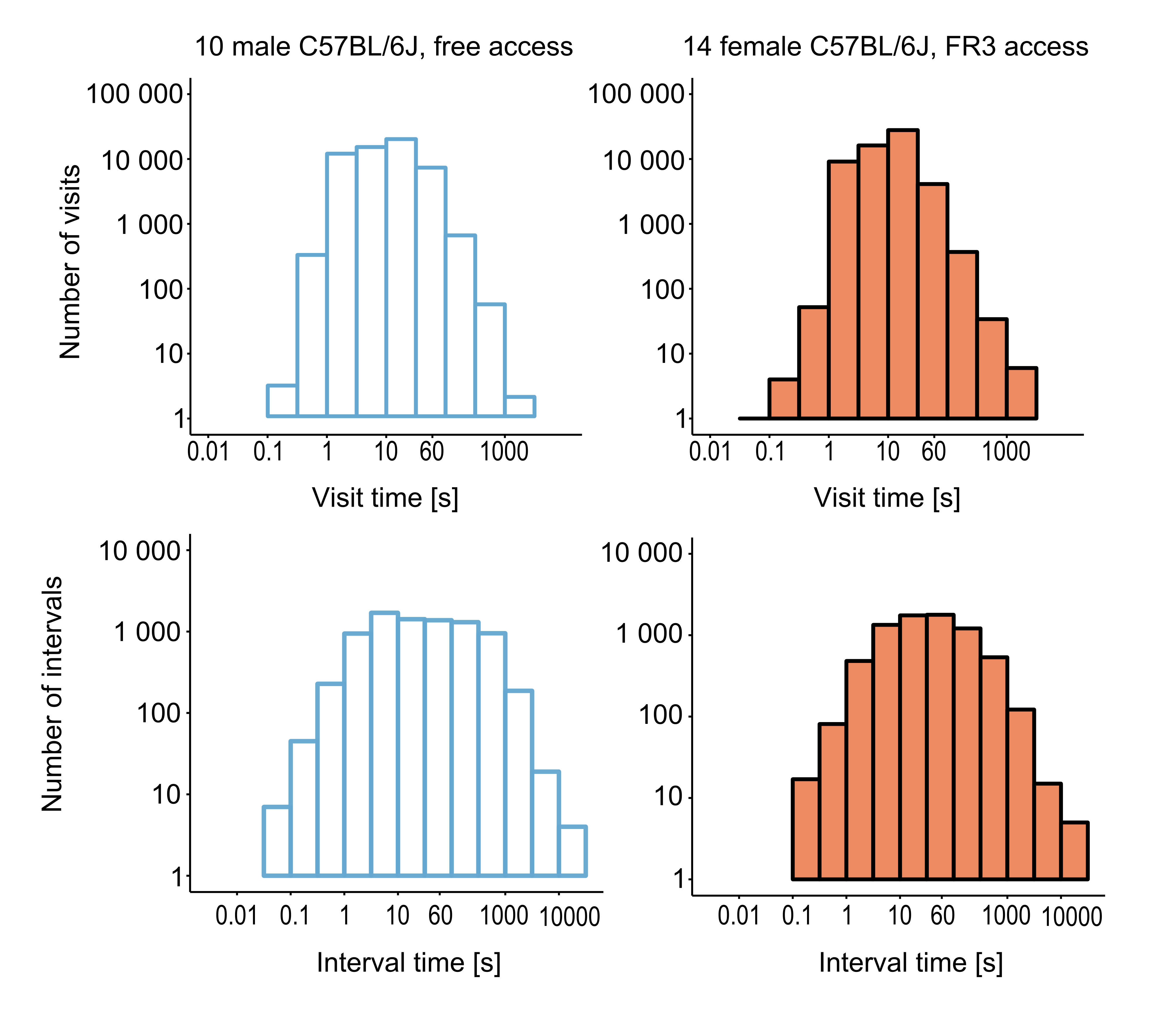

Supplement: Figure S2 — Durations of and intervals between corner visits. The upper two histograms show the distributions of the durations of all corner visits during the 4-week procedure in two representative cohorts of mice (i.e., a group of 10 males and another groups of 14 females). The scales of the axes are logarithmic. The lower two histograms show the distributions of the time intervals between two consecutive visits of an animal to the same corner. The histograms show that most of the visits were > 3 s in duration and that the intervals between consecutive visits were typically > 3 s. While a fraction of the very short times may have resulted from occasional, temporary losses of contact between the cage on the RFID chip in the mouse, such events are unlikely to have significantly contributed to the data. (TIF) [file pone.0096787.s002.tif]

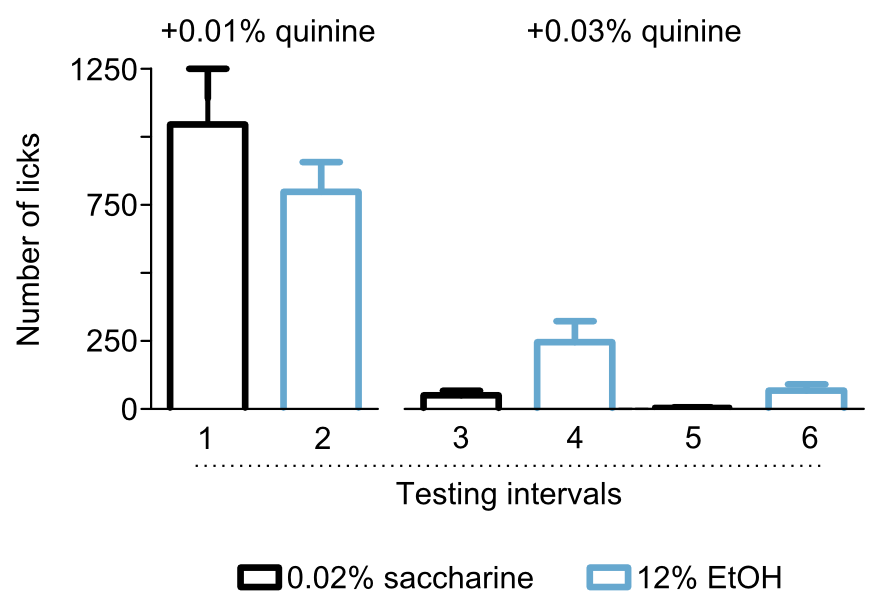

Supplement: Figure S4 — Effects of adulteration with quinine. The bar graphs show mean numbers of licks on bottles with 0.02% saccharin or 12% alcohol adulterated with increasing concentrations of quinine during a single interval of free access. The procedure was carried out over 6 subsequent intervals. Alcohol and saccharin testing was performed during separate intervals, as described in the Methods (see Table S1 for a summary of the experiment). Repeated measures ANOVA F5,59 = 19.35, p<0.001; Tukey's HSD post hoc comparisons of 1 vs. 2, 3 vs. 4 and 5 vs. 6 were not significant. (TIF) [file pone.0096787.s004.tif]

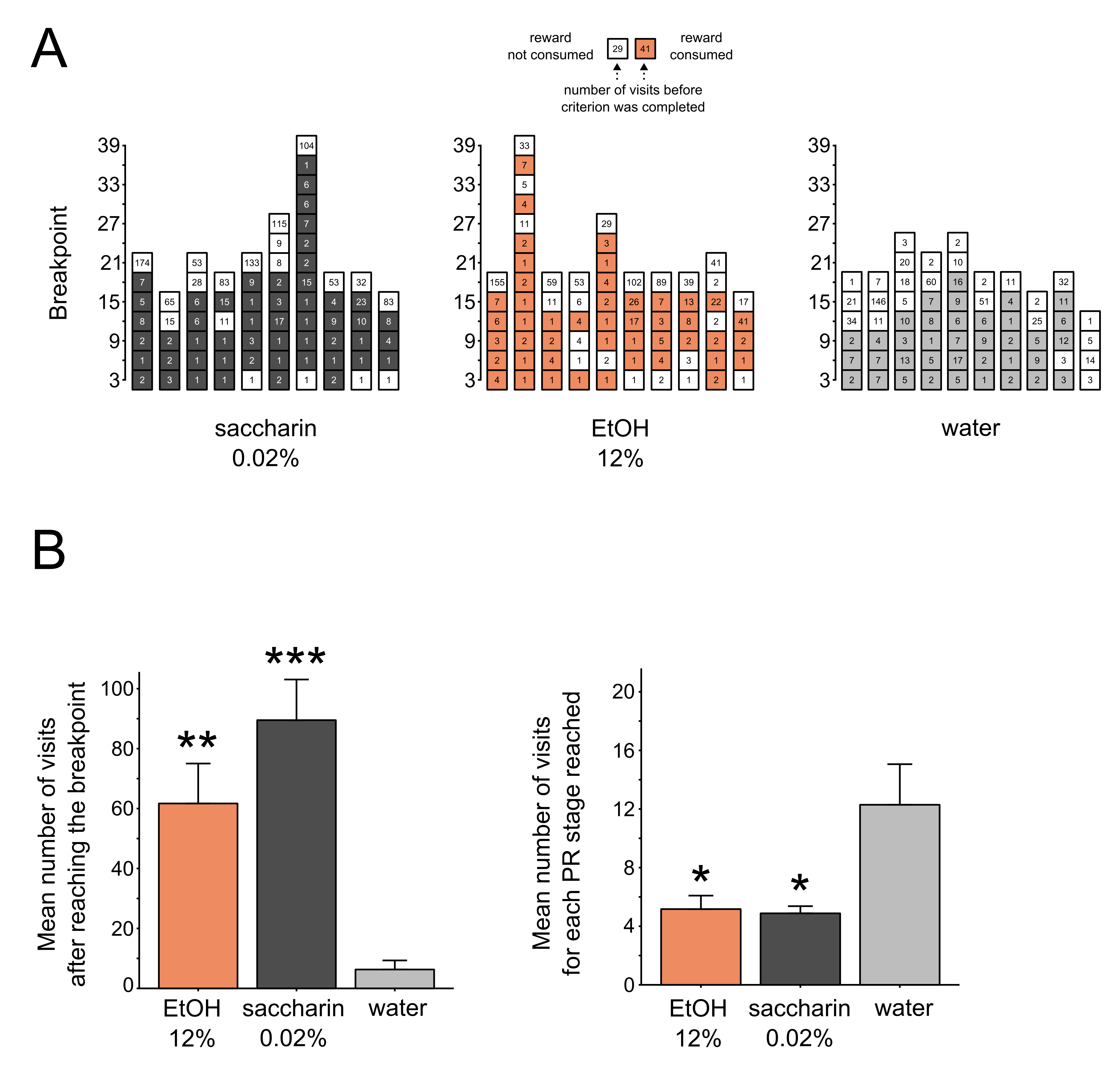

Supplement: Figure S5 — Individual breakpoints of the mice that received PR3 instrumental access to saccharin, alcohol or water. The data shown on the graph represent the same cohorts of mice as shown in Figure 6B. (A) The heights of the bars correspond to the breakpoints of the individual mice. Each box corresponds to a completed PR stage. The filled boxes indicate that the animal licked the bottle after reaching the criterion. The open boxes indicate that no licks were detected (and presumably, the reward was not consumed). The number inside the box indicates the number of visits the mouse performed before reaching the next criterion. The top boxes represent PR criteria that were not reached and are therefore always empty. (B) The graphs show the mean number of visits performed in the “rewarded” corners after the breakpoint was reached (left) and the mean number of visits performed between completion of the PR criteria (right). In both cases, analysis of variance indicated the presence of a significant difference across groups (F2,27 = 14.52, P<0.001 and F2,27 = 6.043 P<0.01, respectively). Significant differences between the mean values of the visits were calculated using Tukey's HSD; “*” corresponds to P<0.05 vs. “water”, “**” P<0.01 and “***” P<0.001. There were no significant differences between the mean numbers of visits by the “alcohol” and “saccharin” groups. (TIF) [file pone.0096787.s005.tif]
