# Supplementary material for: A Model of Alcohol Drinking under an Intermittent Access Schedule Using Group-Housed Mice
Source: PLoS One. 2014 May 7;9(5):e96787. doi: 10.1371/journal.pone.0096787 (PMC4013044; doi:10.1371/journal.pone.0096787)
Supplement: Table S1 — Quinine adulteration schedule. (DOCX) [file pone.0096787.s006.docx]

Table S1. Quinine adulteration schedule.

|  | Interval 1 | Interval 2 | Interval 3 | Interval 4 | Interval 5 | Interval 6 |
| --- | --- | --- | --- | --- | --- | --- |
| Corners I&III | water | Alcohol  12%  +  Quinine 0.01% | Saccharin 0.02%  +  Quinine  0.03% | water | water | Alcohol  12%  +  Quinine 0.03% |
| Corners II&IV | Saccharin 0.02%  +  Quinine  0.01% | water | water | Alcohol  12%  +  Quinine 0.03% | Saccharin 0.02%  +  Quinine  0.03% | water |
